# Study on different isolation technology on the performance of blue micro-LEDs array applications

**DOI:** 10.1186/s11671-024-04047-z

**Published:** 2024-06-13

**Authors:** Shao-Hua Lin, Yu-Yun Lo, Yu-Hsuan Hsu, Chien-Chung Lin, Hsiao-Wen Zan, Yi-Hsin Lin, Dong-Sing Wuu, Ching-Lien Hsiao, Ray-Hua Horng

**Affiliations:** 1 Institute of Electrics, National Yang Ming Chiao Tung University, Hsinchu, 30010 Taiwan, ROC; 2 Department of Photonis, National Yang Ming Chiao Tung University, Hsinchu, 30010 Taiwan, ROC; 3 Graduate Institute of Photonics and Optoelectronics, National Taiwan University, Taipei, 10617 Taiwan, ROC; 4 Department of Applied Materials and Optoelectronic Engineering, National Chi Nan University, Nantou, 54561 Taiwan, ROC; 5 Thin Film Physics Division, Department of Physics, Chemistry and Biology (IFM), Linköping University, 581 83 Linköping, Sweden

**Keywords:** Micro-LED, Pixel size, Ion implantation technology, Electrical isolation

## Abstract

In this study, a 3 × 3 blue micro-LED array with a pixel size of 10 × 10 μm^2^ and a pitch of 15 μm was fabricated on an epilayer grown on a sapphire substrate using metalorganic chemical vapor deposition technology. The fabrication process involved photolithography, wet and dry etching, E-beam evaporation, and ion implantation technology. Arsenic multi-energy implantation was utilized to replace the mesa etching for electrical isolation, where the implantation depth increased with the average energy. Different ion depth profiles had varying effects on electrical properties, such as forward current and leakage currents, potentially causing damage to the n-GaN layer and increasing the series resistance of the LEDs. As the implantation depth increased, the light output power and peak external quantum efficiency of the LEDs also increased, improving from 5.33 to 9.82%. However, the efficiency droop also increased from 46.3 to 48.6%.

## Introduction

Micro-light emitting diodes (micro-LEDs) are considered a key technology for high-resolution displays due to their micron-scale pixel size, high power efficiency, long lifetime, and rapid response speed [1–3]. These advantages enable applications such as self-emission displays [4, 5], flexible devices for wearable applications [6, 7], and high-speed visible light communication [8, 9]. To obtain the high efficient micro-LEDs, the first priority is to obtain the high quality epilayer. In general, GaN-based LEDs are always grown on foreign substrates due to the cost limitation. Therefore, high-density dislocation is obviously induced for the heteroepitaxial GaN film grown on foreign substrates, which determines the device performance. In order to obtain the high quality of GaN epilayers, various techniques, such as facet-controlled epitaxial lateral overgrowth (ELOG), pendeo-epitaxy, serpentine channel, patterned substrate with various shapes, and ion implantation induced high-quality nucleation layer were proposed [10–16]. In 2023, Hong chang Tao et al*.* demonstrated high-quality nucleation in LED epilayers using ion implantation [15]. This method successfully improved the PL intensity and WPE from 30.7% to 37.4% in LED performance. Furthermore, two-dimensional (2D) group-III nitride materials and devices were also developed [16].

Not only the high quality epilayer, the complex fabrication process for high-resolution displays, including thin film deposition, etching, mass-transfer, and circuit bonding processes, also exists the challenges [17, 18]. Moreover, downsizing pixel sizes reduces the efficiency of micro-LEDs due to damage to the mesa sidewalls induced by non-radiative recombination centers caused by the dry etch process, particularly using inductively coupled plasma reactive-ion etching (ICP-RIE) [19–21]. Passivation layers are commonly used to repair these sidewall damages [22–25]. Additionally, chip sizes are prone to distortion by ICP-RIE, leading to trapezoidal shapes and further reducing the emission area. To address the bottleneck in device performance, a new fabrication strategy for micro-LEDs using ion implantation has been introduced [26–29]. By implanting specific ions into GaN-based epitaxial layers, lattice damage occurs in the target region, enabling the formation of high resistivity regions and even electrical isolation [30]. Generally, higher implantation energy and the use of lighter mass elements increase implantation depth. However, using excessively light mass elements such as hydrogen or helium may result in poor thermal stability [31, 32], increased leakage current, and degraded pixelation contrast due to ion scattering. Low-energy injection can mitigate unintentional lateral spreading, while high-energy injection ensures sufficient injection depth. Furthermore, multi-energy implantation is often employed to achieve uniform spatial distribution [33] for optoelectronic devices. However, multi-energy implantation has been rarely utilized in micro-LED applications. Therefore, ion implantations with multi-energy injections are proposed to suppress lateral spreading while maintaining the same injection depth. Traditionally, the epitaxial layer undergoes thermal annealing to recover lattice damage caused by multi-energy injections. However, for micro-LED applications, thermal treatment after multi-energy implantation may not be necessary. Hence, it is important to evaluate whether high-energy or low-energy implantation is performed first. Moreover, an inappropriate combination of implant energy and dosage may adversely affect device performance [34]. In this work, 3 × 3 GaN-based blue micro-LED arrays with a pixel size of 10 × 10 μm^2^ fabricated by multi-energy implantation using arsenic ions (As^+^) were demonstrated. The effect of multi-energy implantation, whether high energy first or low energy first, on the performance of micro-LEDs was studied. The dosage with different implantation energies was also investigated. The electrical and photoelectric properties of the micro-LED array were measured and discussed, along with the simulation and analysis of ion depth profiles for each sample.

## Experimental methods

**Fig. 1:**
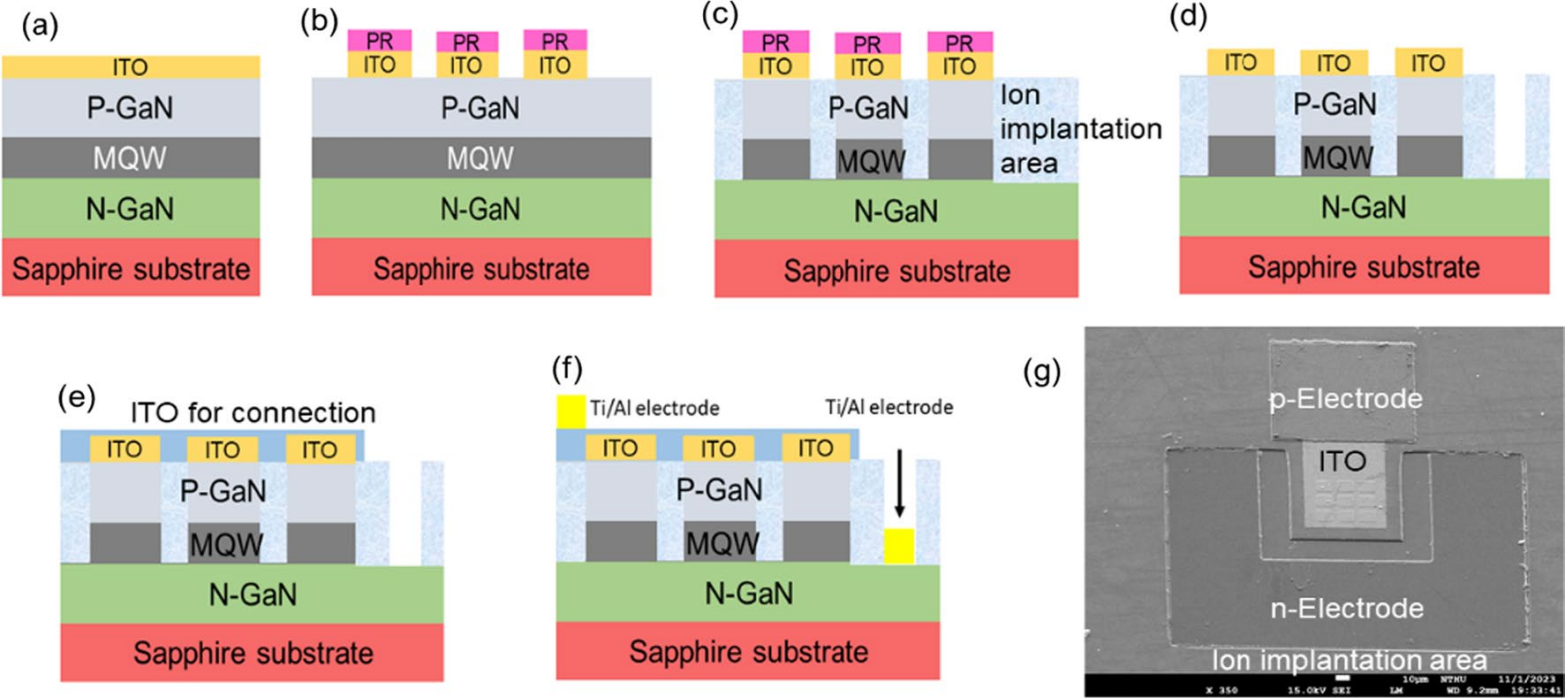
Fabrication process flow diagram **a** ITO deposited on the LED spilayers, **b** defining the 3 × 3 array pattern ITO layer with 10 × 10 μm^2^ area, **c** ion implantation processing, **d** etching to exposure n-GaN layer, **e** deposition of the second ITO layer for 3 × 3 array connection, **f** deposition of n and p electrodes, and **g** top view SEM image of final micro-LED array

The InGaN/GaN blue LED epitaxy was grown on a 4-inch sapphire substrate using metalorganic chemical vapor deposition. The LED epitaxial structure, from surface to substrate, consists of a 100 nm-thick layer of indium-tin oxide (ITO), a 400 nm-thick p-GaN, 300 nm-thick multiple quantum wells (MQWs), and a 4 μm-thick n-GaN, as depicted in Fig. 1a. Following the deposition of the 100 nm-thick ITO layer, a thermal annealing process was conducted to achieve Ohmic contact. Subsequently, a 10 × 10 μm^2^ pixel array with a pitch of 15 μm, arranged in a 3 × 3 pattern, was defined on the ITO layer and etched using an HCl-FeCl_3_ solution, as shown in Fig. 1b. Ion implantation was carried out after wet etching of the ITO layer, with a masking step using a 4 μm-thick photoresist layer, as illustrated in Fig. 1c. The ions with different energy and dosages were carried out with a 7° tilt-angle using ion implanter at room temperature. Next, a portion of the p-GaN and MQW layers was dry-etched using inductively coupled plasma reactive-ion etching (ICP-RIE) with a Cl_2_-BCl_3_ gas mixture to expose the n-GaN layer for the n-metal contact window, as shown in Fig. 1d. Following the etching of the p-GaN layer, a second layer of ITO was deposited using a sputter system, followed by a lift-off process to retain the top p-region connection, as depicted in Fig. 1e. This additional ITO layer served to prevent the pixel light from being obstructed by the p-metal layer. Finally, both p-metal and n-metal (Ti/Al, 40/400 nm) electrodes were deposited using an e-gun evaporation system, as shown in Fig. 1f. The top-view image of the final micro-LED array was captured using a scanning electron microscope (SEM), as presented in Fig. 1g.

The Stopping and Range of Ions in Matter (SRIM) software was utilized to simulate ion implantation in this study. The target material was specified as GaN, with a density of 6.15 g/cm^3^. Since multi-energy implantation was investigated in this research, the simulation involved performing separate simulations for single-energy implantation with different implant energies, which were then superimposed to generate the final result. To examine the effect of multi-energy implantation on the performance of micro-LEDs, samples A and B were subjected to implantation with low energy first and high energy first, respectively. It was noteworthy that the dosages for implantation energies of 10, 20, 30, and 40 keV were consistent at 1 × 10^14^ cm⁻^2^. The total dosages are 4 × 10^14^ cm⁻^2^ in this study. Sample C was configured with different dosages compared to sample B but employed almost the same multi-energy implantation scheme (total dosages are 3.9 × 10^14^ cm⁻^2^). The implantation parameters for each sample are detailed in Table 1. The experimental depth profiles of the implanted samples were analyzed using secondary ion mass spectrometry (SIMS). The electrical characteristics of the micro-LEDs were assessed using an Agilent 4155B semiconductor parameter analyzer, while their optoelectrical properties were measured using a multi-function power meter KEITHLEY 2400 equipped with integrating sphere systems. Surface images of the processed micro-LED arrays were captured using a scanning electron microscope (SEM).

**Table 1:** As Implantation parameters of each sample in this study, which the multi-energy implantation was applied in the order of (i) → (ii) → (iii) → (iv)

Sample	Implantation parameters
A	(i) 10 keV, 1 × 10^14^ cm^−2^, (ii) 20 keV, 1 × 10^14^ cm^−2^ (iii) 30 keV, 1 × 10^14^ cm^−2^, (iv) 40 keV, 1 × 10^14^ cm^−2^
B	(i) 40 keV, 1 × 10^14^ cm^−2^, (ii) 30 keV, 1 × 10^14^ cm^−2^ (iii) 20 keV, 1 × 10^14^ cm^−2^, (iv) 10 keV, 1 × 10^14^ cm^−2^
C	(i) 40 keV, 3 × 10^14^ cm^−2^, (ii) 30 keV, 3 × 10^13^ cm^−2^ (iii) 20 keV, 3 × 10^13^ cm^−2^, (iv) 10 keV, 3 × 10^13^ cm^−2^

## Results and discussion

### Secondary ion mass spectroscopy analysis

**Fig. 2:**
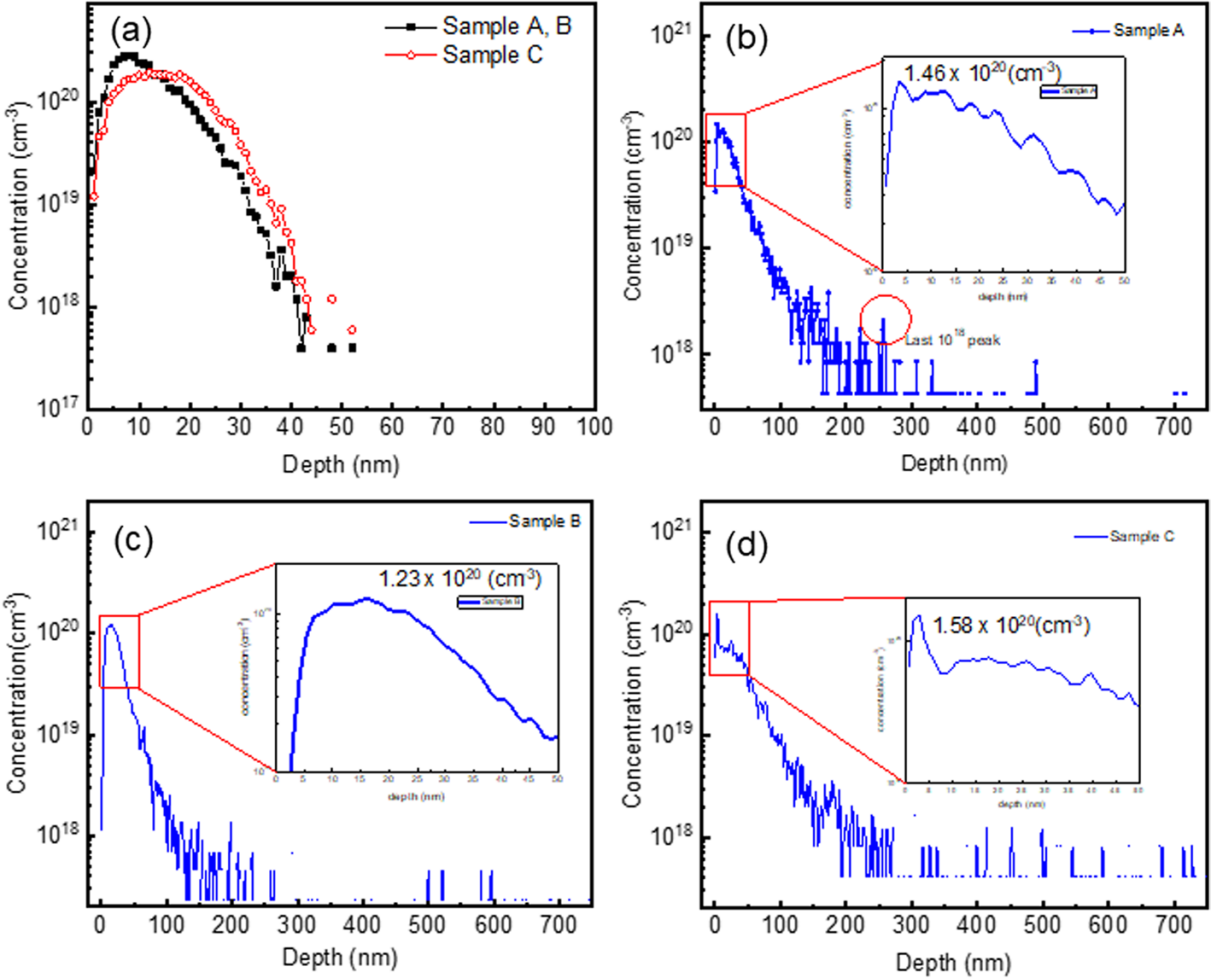
**a** Simulated Depth profile of each sample and depth profiles of As concentration measured by SIMS **b** sample A, **c** sample B and **d** sample C

The depth profile of each sample simulated by SRIM is shown in Fig. 2a. There were no differences in concentration profiles between Samples A and B in the simulation, as the dosages and energies were identical, and the results of multi-energy implantation were derived from superimposing the results of single-energy implantation. Sample C exhibited a more uniform concentration profile compared to Samples A and B, attributed to the different dosages employed. The maximum implantation depth (by simulation) for Samples A, B, and C was similar, approximately 10 nm, as their maximum implantation energies were all 40 keV.

The experimental depth profiles of As concentration in each sample were analyzed by SIMS and depicted in Fig. 2b–d. In sample A, the concentration of As in the near-surface region, approximately 5 nm deep, was measured at 1.46 × 10^20^ cm^−3^, with a maximum detectable implantation depth of about 500 nm, as shown in Fig. 2b. Conversely, in sample B, the peak concentration of As, occurring at a depth of 15 nm, was approximately 1.23 × 10^20^ cm^−3^, with the maximum detectable implantation depth increasing to 600 nm, as illustrated in Fig. 2c. This shift in As distribution towards deeper regions of the material was attributed to the altered order of implantation in the multi-energy ion implantation process. Specifically, in sample A, where the highest energy dose (40 keV) was implanted last, there was less of a channeling effect during the implementation of the highest energy dose, as the lattice in the shallow region had already been damaged by the earlier, lower energy dose. This suggests that for deeper isolation requirements, multi-energy implantation should prioritize the use of high energy first. It was worthy to mention that the highest concentration was obtained in Sample C due to the highest dosage with the highest implant energy first.

It was observed that the As concentration in sample C decreased at a slower rate compared to sample B, despite both samples following the same multi-energy sequence. Additionally, more concentration peaks were evident at a depth of 300 nm, with the maximum detectable implantation depth reaching 700 nm for sample C. This phenomenon can be attributed to the higher dosage of 40 keV implanted in sample C, allowing more ions to penetrate deeper into the material with higher energy. Furthermore, the higher concentration peak near the surface of sample C, potentially caused by backscattered ions, suggests that ions with low energy may scatter back due to lattice damage from high energy implantation. Consequently, this leads to a more uniform distribution of As. These findings align with the results obtained from the SRIM simulation, confirming the effectiveness of the multi-energy implantation strategy in achieving a desired ion distribution profile. Overall, deepest penetration occurred in the parameters of Sample C. Although it is hard to directly demonstrate the true As concentration levels at the depth, and they could just related to the edge sputtering effect during the SIMS testing. The signal distribution tendency of the implantation depth increase from sample A to B and then to C can be obtained. This behavior will also be demonstrated by electrical properties of micro-LEDs samples A, B and C and will be discussed later.

### Electrical properties

**Fig. 3:**
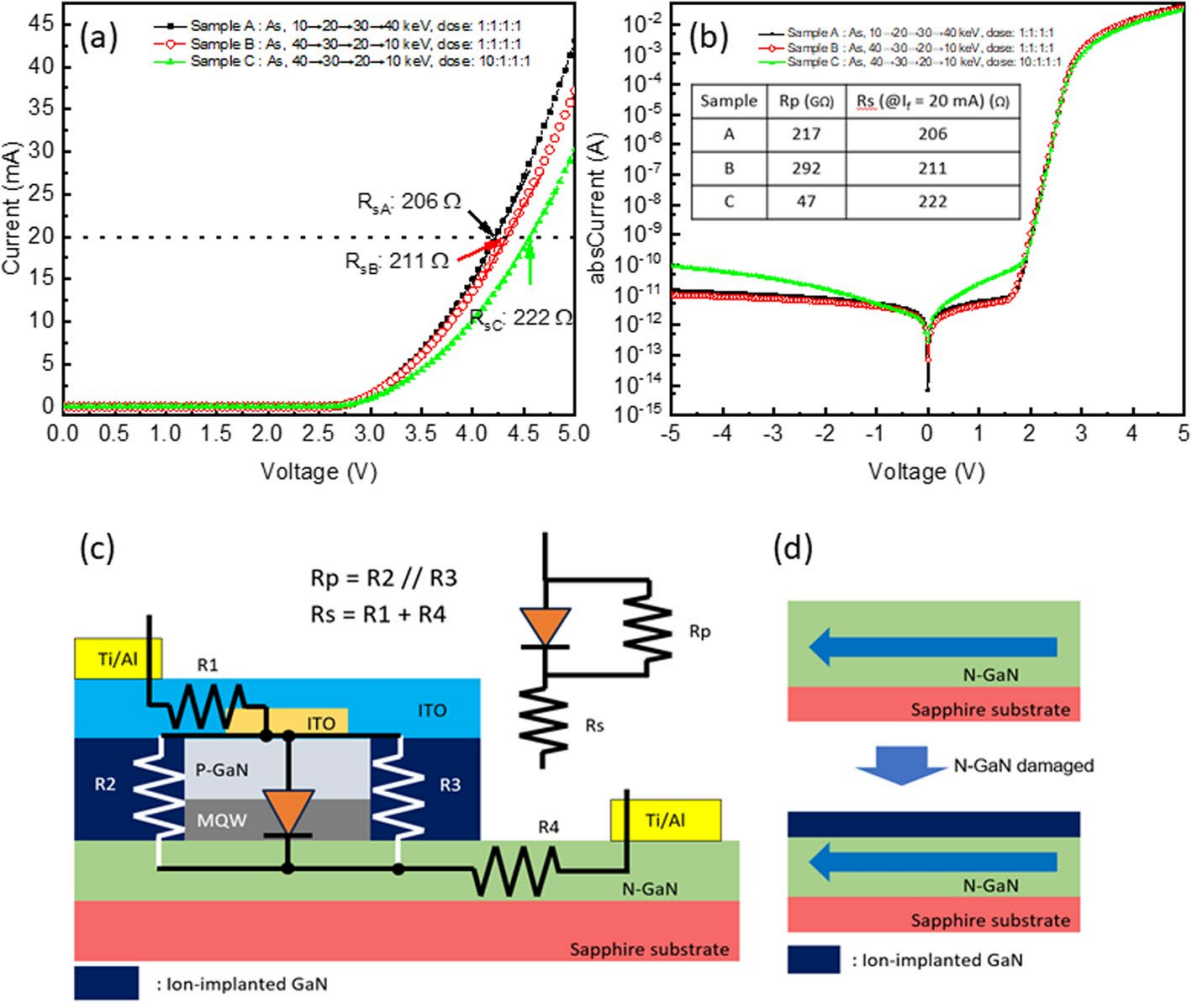
**a** Forward IV curves from 0 to 5 V of each sample, **b** IV curves under semi-log scale from − 5 to 5 V of each sample, **c** Schematice of micro-LED structurewith the Rs and Rp, and **d** Cross section reduction of damaged n-GaN. The inset Table of (**b**) presented the R_s_ and R_p_ calculated from IV curves

It was crucial to evaluate whether these three implantation parameters could fulfill the mesa function and their effect on the performance of micro-LEDs. The forward current as a function of voltage (I-V) curves and IV curves under semi-log scale of each sample were measured, and the results are depicted in Fig. 3a, b. It was observed that the forward voltage increased from sample A, B, to C when operated at the same forward current. The corresponding series resistances R_sA_, R_sB_, and R_sC_ were 206, 211, and 222 Ω, respectively. This trend could be attributed to the increasing implantation depth, resulting in a decrease in the conductive path. This observation was further supported by the results of SIMS. As the implantation depth increased, the forward voltage also increased from sample A, B, to C, indicating that the n-GaN layer was more severely damaged, leading to an increase in the series resistance of the device from 206 Ω to 222 Ω under a 20 mA current injection, respectively, owing to deeper ion implantation [35]. The schematic representation of series resistance, R_s_, and parallel resistance, R_p_, in the device structure is shown in Fig. 3c. The R_s_ primarily comprised the parasitic resistance of n-GaN and ITO, while R_p_ existed in parallel to the LED. As illustrated in Fig. 3d, a damaged n-GaN layer could reduce the effective cross-sectional area of the current path, resulting in a higher R_s_. The R_s_ and R_p_ calculated from IV curves are presented in the inset Table of Fig. 3b. Clearly, sample C, implanted using high energy first with high dosage, exhibited the deepest depth and highest R_s_. Furthermore, due to the highest dosage first (3 × 10^14^ cm^−2^) in sample C, it could also damage the MQW and result in the lowest R_p_.

Sample B exhibits a smaller leakage current than sample A due to the improved isolation resulting from deeper implantation using high implantation energy first, while sample C shows a significantly larger leakage. This discrepancy can be attributed to the deeper average implantation depth in sample C, which implies a lower average As concentration under the same total dosage, resulting in poorer isolation and smaller damage to the material, consequently leading to a smaller Rp, as depicted in Fig. 3b. When the device is reverse-biased, the incomplete isolation of GaN beside the LED forms a leakage path, resulting in a larger leakage current.

### Optoelectric properties

**Fig. 4:**
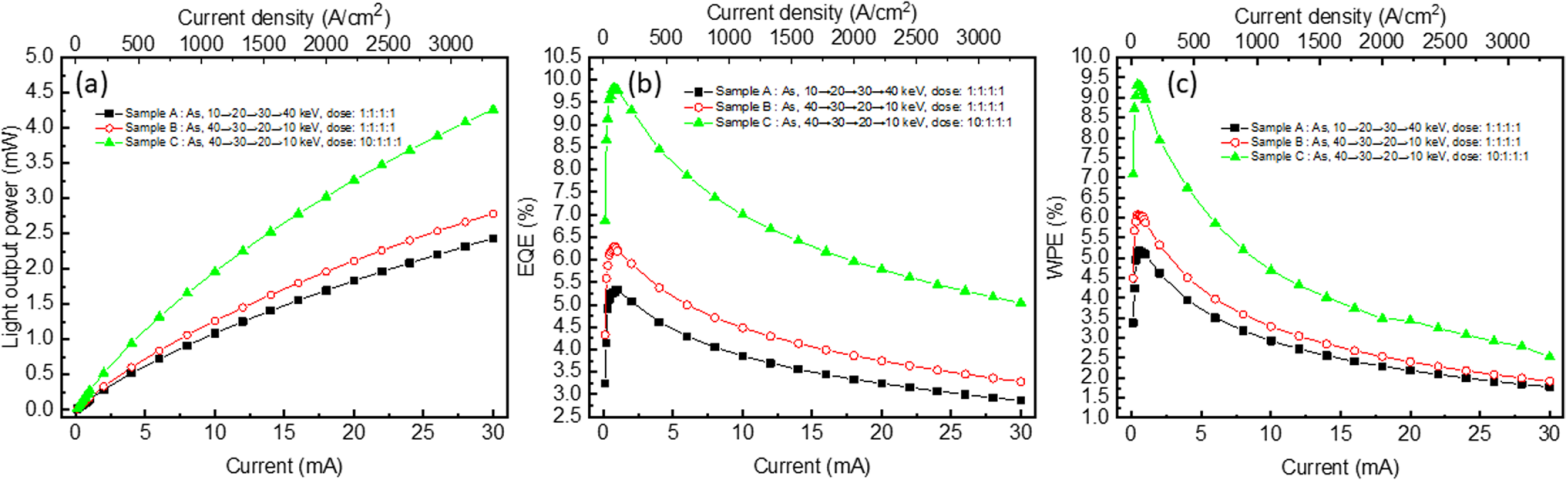
**a** Light output power, **b** EQE, and **c** WPE as functions of injection current for samples A, B and C micro-LEDs arrays

Although there are slight variations in the I-V characteristics among samples A, B, and C, the most crucial aspect is the optoelectronic performance of these micro-LED arrays. Light output power (LOP), external quantum efficiency (EQE), and wall-plug efficiency (WPE) measurements were conducted using an integrating sphere, and the results are depicted in Fig. 4a–c, respectively. The LOP of all samples increases as the injection current rises, albeit with a decreasing slope, indicating efficiency droops. As the implantation depth increases from sample A to B and then to C, the LOP, EQE, and WPE exhibit a monotonic increase. The peak EQE values are 5.33%, 6.28%, and 9.82%, while the peak WPE values are 5.17%, 6.07%, and 9.31%, respectively, for samples A, B, and C. The efficiency droop under an injection current of 30 mA is 46.3%, 47.7%, and 48.6% for samples A, B, and C, respectively. The EQE of micro-LEDs is closely related to defects in the LED epilayers. Defects in the material form non-radiative recombination centers, and carriers trapped by these centers tend to undergo non-radiative recombination, known as Schockley-Read-Hall (SRH) recombination, thereby lowering the EQE. Conversely, as the EQE is reduced by SRH recombination, Auger recombination is also suppressed, playing a crucial role in efficiency droop [19, 36]. Therefore, the results suggest that the increase in EQE is attributed to fewer defects in the material, resulting in fewer carriers undergoing SRH recombination and a more pronounced efficiency droop. It is noteworthy that all samples were fabricated using the same epilayers, indicating that the different performance of the micro-LED array resulted from the ion implantation parameters. After ion implantation, the lattice is damaged by high-energy ions, and point defects such as vacancies or interstitial defects exist in the material. Carriers trapped by these recombination centers undergo non-radiative recombination, thereby lowering the device efficiency. With the implantation depth increasing, using high ion implantation energy first with high dosage, a uniform concentration profile (as observed in sample C) is achieved. This results in the best isolation of the MQW layer between pixels, and fewer carriers flow through incompletely isolated material, leading to the best light output power and EQE performance, as shown in Fig. 4a, b. Although sample C exhibits slightly higher Rs, it appears to have no effect on the performance. Sample C exhibits the highest WPE among these micro-LED arrays.

**Fig. 5:**
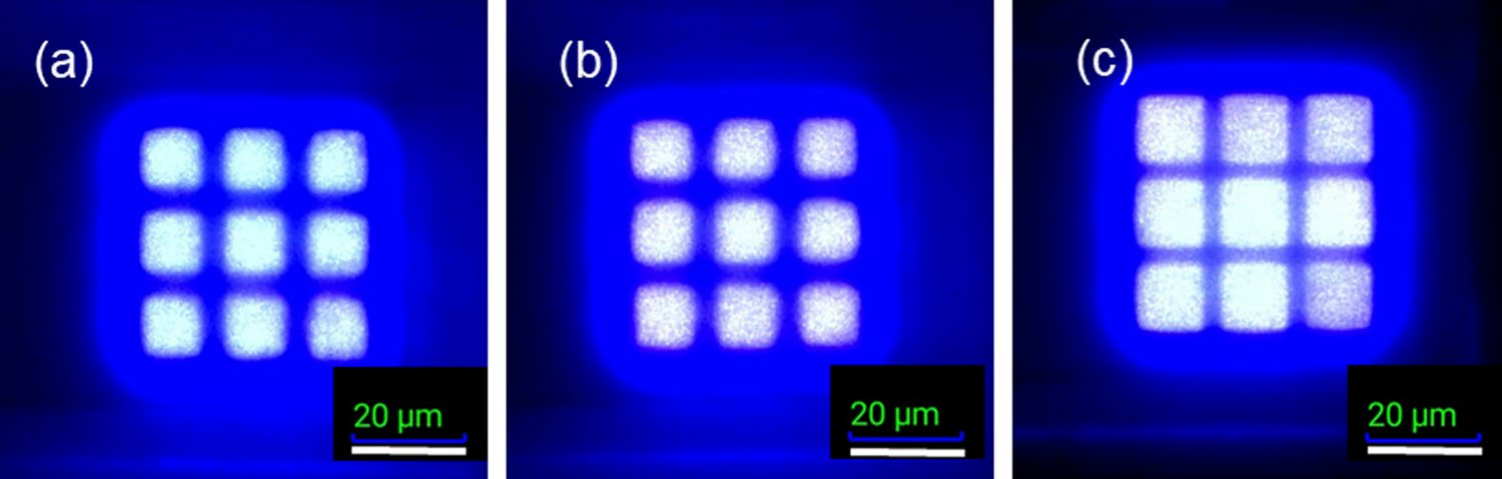
EL images of micro-LED arrays under 5 mA injection of **a** sample A, **b** sample B and **c** sample C

The electroluminescence (EL) images of samples A, B, and C under an injection current of 5 mA are depicted in Fig. 5. Evidently, sample C exhibits the brightest array. Despite all samples having the 5 μm channel ion implanted, the channel regions differed. The dark region of sample A was the widest, followed by sample B, with sample C having the narrowest dark region. It could be resulted that a highest dosages of 40 keV were used in Samples C. Isolation of samples C would be more effective in shallow regions compared to those of Sample A and B. It resulted in high current density being injected in sample C which presented the most brightness and narrowest dark region. This demonstrates that the As ions were driven deep enough to achieve isolation between LED pixels under a forward current of 5 mA. Moreover, the non-uniform image in sample C was resulted from the CCD image sensor saturation.

**Fig. 6:**
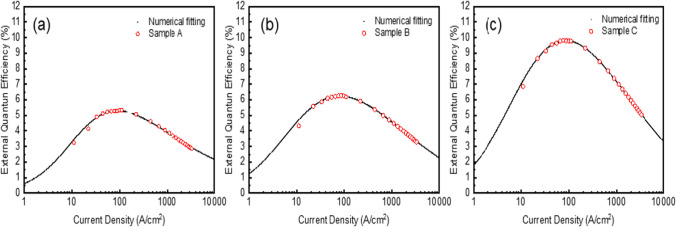
Measured EQE versus current density with numerically fitted curves of **a** Sample A, **b** Sample B, and **c** Sample C

From the measured EQE (similar to what we saw in Fig. 4b), the Shockley–Read–Hall (SRH) coefficients of each ion-implanted devices can be extracted [37, 38]. The non-radiative recombination in a micro LED can be found in the SRH and Auger recombinations [39]. While the SRH process dominates at the low current levels, the Auger recombination can be effective in the high current injection. Together with the radiative recombination (governed by the bi-molecular coefficient), the so-called ABC model can be used for the device evaluation, particularly when the non-radiative recombination could be detrimental to the performances of the micro-scale devices [39]. Combining these three factors, we can use the following expressions to theoretically calculate or fit the EQE results [37, 38]:

1 $${J}_{total}=\frac{qt\left(An+B{n}^{2}+C{n}^{3}\right)}{\left(1-\beta n\right)}$$

2 $$EQE=\frac{{\eta }_{LEE}\left(1-\beta n\right)B{n}^{2}}{\left(An+B{n}^{2}+C{n}^{3}\right)}$$

where J_total_ is the total current density injected into the device, η_LEE_ is the light extraction coefficient which can be treated as a constant in the device, and the parameters of A, B, and C corresponds to the SRH, the bi-molecular, and the Auger recombination coefficients in the devices. An additional factor β is a fitting parameter related to the leakage of carriers [30]. If there is negligible leakage, the value of β can be set as zero and the whole expression becomes the traditional ABC model [40]. Figure 6a–c demonstrate both the measured and the calculated (fitted) results from various ion-implantation conditions. The extracted SRH coefficients are listed in Table 2.

**Table 2:** The extracted SRH coefficients from various ion-implantation conditions

Samples	Sample A	Sample B	Sample C
SRH coefficient (sec^−1^)	6.85 × 10^6^	3.72 × 10^6^	3.99 × 10^6^

The sample A has the highest SRH coefficient among the three cases and thus its EQE is also the lowest. Meanwhile, the samples B and C have much lower SRH coefficients and their EQE values are also higher (EQE_max_ = 9.82% for sample C), indicating that they are the preferred choice of the ion-implantation. The difference between sample B and sample C is mainly on the dosage of the implantation, which could affect the disordering brought by the implanted atoms, and the damages in the crystal. We can observe this phenomenon in their reverse leakage currents in Fig. 3b, where the detected reverse current is 8.94 × 10^−11^ A at − 5V for the sample C and 1.4 × 10^−11^ A for the sample B. But the effective depth of the implantation in sample C is greater than that of the sample B and this could provide a better current injection during the operation and lead to a better EQE in Fig. 6.

## Conclusion

In this study, 10 × 10 μm^2^, 3 × 3 blue micro-LED arrays were fabricated using ion implantation technology, and the external quantum efficiency (EQE) of the LEDs was improved with an optimized multi-energy ion implantation with multiple dosages. Based on the results from secondary ion mass spectrometry (SIMS) analysis and electroluminescence (EL) images, arsenic (As) implantation was able to penetrate the n-GaN layer to a depth of 700 nm, achieving isolation between pixels. It was observed that the electrical properties of micro-LEDs, such as series resistance and parallel resistance, were influenced by the ion implantation process. Incomplete isolation may result in a smaller parallel resistance, while deep implantation damaging the n-GaN layer may lead to a larger series resistance. The peak EQE of the micro-LEDs increased from 5.33% to 9.82%, and the peak wall-plug efficiency (WPE) increased from 5.17% to 9.31% as the implantation depth increased, respectively. This improvement in efficiency can be attributed to better isolation of the multiple quantum wells (MQWs), resulting in fewer carriers undergoing non-radiative recombination in the implanted epilayer. These findings demonstrate that the performance of micro-LEDs is closely related to ion implantation process parameters and the ion depth profile.

## Data Availability

The datasets used and/or analyzed during the current study are available from the corresponding author on reasonable request.

## References

[1] Behrman K, Kymissis I (2022). Micro light-emitting diodes. Nat Electron.

[2] Wong MS, Nakamura S, DenBaars SP (2019). Review—progress in high performance III-nitride micro-light-emitting diodes. ECS J Solid State Sci Technol.

[3] Wu Y, Ma J, Su P, Zhang L, Xia B (2020). Full-color realization of micro-LED displays. Nanomaterials.

[4] Zhang X, Li P, Zou X, Jiang J, Yuen SH, Tang CW, Lau KM (2019). Active matrix monolithic LED micro-display using GaN-on-Si epilayers. IEEE Photon Technol Lett.

[5] Chen CJ, Chen HC, Liao JH, Yu CJ, Wu MC (2019). Fabrication and characterization of active-matrix 960×540 blue GaN-based micro-LED display. IEEE J Quantum Electron.

[6] Lee HE, Shin JH, Lee SH, Lee JH, Park SH, Lee KJ (2019). Flexible micro light-emitting diodes for wearable applications. Light-Emitting Devices Mater Appl.

[7] Hu LH, Choi J, Hwangbo S, Kwon D, Jang B, Ji S, Kim JH, Han SK, Ahn JH (2022). Flexible micro-LED display and its application in Gbps multi-channel visible light communication. npj Flex Electron.

[8] Wei ZX, Wang L, Li ZH, Chen CJ, Wu MC, Wang L, Fu HY (2023). Micro-LEDs illuminate visible light communication. IEEE Commun Mag.

[9] Lan HY, Tseng IC, Lin YH, Lin GR, Huang DW, Wu CH (2020). High-speed integrated micro-LED array for visible light communication. Opt Lett.

[10] Khan MSA, Liao H, Yu G, Iqbal I, Lei M, Lang R, Mi Z, Chen H, Zong H, Hu X (2021). Reduction of threading dislocations in GaN grown on patterned sapphire substrate masked with serpentine channel. Mater Sci Semicond Process.

[11] Zhao Y, Xu S, Zhang J, Zhang C, Li P, Lin Z, Zhang Y, Zhou H, Wang Z, Peng R, Fan X, Du J, Hao Y. Optical properties evolution of GaN film grown via lateral epitaxial overgrowth. Appl. Surf. Sci. 2020;513(30):145816. 10.1016/j.apsusc.2020.145816

[12] Gao H, Yan F, Zhang Y, Li J, Zeng Y, Wang G. Enhancement of the light output power of InGaN/GaN light-emitting diodes grown on pyramidal patterned sapphire substrates in the micro- and nanoscale. J Appl Phys (Melville, NY, US) 2008;103(1)L014314.

[13] Chao SH, Yeh LH, Wu RT, Kawagishic K, Hsu SC (2020). Novel patterned sapphire substrates for enhancing the efficiency of GaN-based light-emitting diodes. RSC Adv.

[14] Hiramatsu K, Nishiyama K, Onishi M, Mizutani H, Narukawa M, Motogaito A, Miyake H, Iyechika Y, Maeda T (2000). Fabrication and characterization of low defect density GaN using facet-controlled epitaxial lateral overgrowth (FACELO). J Cryst Growth.

[15] Tao H, Xu S, Zhang J, Su H, Gao Y, Zhang Y, Zhou H, Hao Y (2023). Improved crystal quality and enhanced optical performance of GaN enabled by ion implantation induced high-quality nucleation. Opt Express.

[16] Wang W, Jiang H, Li L, Li G (2021). Two-dimensional group-III nitrides and devices: a critical review. Rep Prog Phys.

[17] Zhu G, Liu Y, Ming R, Shi F, Cheng M (2022). Mass transfer, detection and repair technologies in micro-LED displays. Sci China-Mater.

[18] Zhou XJ, Tian PF, Sher CW, Wu J, Liu HZ, Liu R, Kuo HC (2020). Growth, transfer printing and colour conversion techniques towards full-colour micro-LED display. Prog Quantum Electron.

[19] Olivier F, Daami A, Licitra C, Templier F (2017). Shockley-read-Hall and Auger non-radiative recombination in GaN based LEDs: a size effect study. Appl Phys Lett.

[20] Smith JM, Ley R, Wong MS, Baek YH, Kang JH, Kim CH, Gordon MJ, Nakamura S, Speck JS, DenBaars SP (2020). Comparison of size-dependent characteristics of blue and green InGaN microLEDs down to 1 μ m in diameter. Appl Phys Lett.

[21] Bulashevich KA, Karpov SY (2016). Impact of surface recombination on efficiency of III-nitride light-emitting diodes. Phys Status Solidi-Rapid Res Lett.

[22] Yu JC, Tao T, Liu B, Xu FF, Zheng Y, Wang X, Sang YM, Yan Y, Xie ZL, Liang SH, Chen DJ, Chen P, Xiu XQ, Zheng YD, Zhang R (2021). Investigations of sidewall passivation technology on the optical performance for smaller size GaN-based micro-LEDs. Crystals.

[23] Park J, Baek W, Geum DM, Kim S (2022). Understanding the sidewall passivation effects in AlGaInP/GaInP micro-LED. Nanoscale Res Lett.

[24] Chen D, Wang Z, Hu FC, Shen C, Chi N, Liu W, Zhang DW, Lu HL (2021). Improved electro-optical and photoelectric performance of GaN-based micro-LEDs with an atomic layer deposited AlN passivation layer. Opt Express.

[25] Son KR, Murugadoss V, Kim KH, Kim TG (2022). Investigation of sidewall passivation mechanism of InGaN-based blue microscale light-emitting diodes. Appl Surf Sci.

[26] Noor Elahi AM, Xu J (2020). Electrical and optical modeling of gap-free III-nitride micro-LED arrays. AIP Adv.

[27] Xu F, Tan Y, Xie Z, Zhang B (2021). Implantation energy- and size-dependent light output of enhanced-efficiency micro-LED arrays fabricated by ion implantation. Opt Express.

[28] Ye JY, Peng YY, Luo CL, Wang HA, Zhou XT, Guo TL, Sun J, Yan Q, Zhang YA, Wu CX (2023). Pixelation of GaN based Micro-LED arrays by tailoring injection energy and dose of fluorine ion implantation. J Lumines.

[29] Lin XD, Hsu YH, Lin YH, Horng RH. Ion implantation effect on the performance of micro-light emitting diodes array. In 2023 IEEE photonics conference, IPC. 10.1109/IPC57732.2023.10360513

[30] Pearton SJ (1990). Ion implantation for isolation of III-V semiconductors. Mater Sci Rep.

[31] Binari SC, Dietrich HB, Kelner G, Rowland LB, Doverspike K, Wickenden DK (1995). H, He, and N implant isolation of n-type GaN. J Appl Phys.

[32] Pearton SJ, Vartuli CB, Zolper JC, Yuan C, Stall RA (1995). Ion implantation doping and isolation of GaN. Appl Phys Lett.

[33] Komarov FF, Kamyshan AS, Mironov AM, Lagutin AE, Martynov IS (2001). Formation of device isolation in GaAs with polyenergetic ion implantation. Vacuum.

[34] Park J, Choi JH, Kong K, Han JH, Park JH, Kim N, Lee E, Kim D, Kim J, Chung D, Jun S, Kim M, Yoon E, Shin J, Hwang S (2021). Electrically driven mid-submicrometre pixelation of InGaN micro-light-emitting diode displays for augmented-reality glasses. Nat Photon.

[35] Hsu YH, Wang CH, Lin XD, Lin YH, Wuu DS, Horng RH (2023). Improved electrical properties of micro light-emitting diode displays by ion implantation technology. Discov Nano.

[36] Liu ZQ, Wei TB, Guo EQ, Yi XY, Wang LC, Wang JX, Wang GH, Shi Y, Ferguson I, Li JM (2011). Efficiency droop in InGaN/GaN multiple-quantum-well blue light-emitting diodes grown on free-standing GaN substrate. Appl Phys Lett.

[37] Li YY, Lin FZ, Chi KL, Weng SY, Lee GY, Kuo HC, Lin CC (2022). Analysis of size-dependent quantum efficiency in AlGaInP Micro–light-emitting diodes with consideration for current leakage. IEEE Photon J.

[38] Lee TY, Huang YM, Chiang H, Chao CL, Hung CY, Kuo WH, Fang YH, Chu MT, Wu CI, Lin CC, Kuo HC (2022). Increase in the efficiency of III-nitride micro LEDs by atomic layer deposition. Opt Express.

[39] Lin GB, Meyaard D, Cho J, Schubert EF, Shim H, Sone C (2012). Analytic model for the efficiency droop in semiconductors with asymmetric carrier-transport properties based on drift-induced reduction of injection efficiency. Appl Phys Lett.

[40] Huang HH, Huang SK, Tsai YL, Wang SW, Lee YY, Weng SY, Kuo HC, Lin CC (2020). Investigation on reliability of red micro-light emitting diodes with atomic layer deposition passivation layers. Opt Express.

